# Myosin-5 varies its step length to carry cargo straight along the irregular F-actin track

**DOI:** 10.1073/pnas.2401625121

**Published:** 2024-03-20

**Authors:** Adam Fineberg, Yasuharu Takagi, Kavitha Thirumurugan, Joanna Andrecka, Neil Billington, Gavin Young, Daniel Cole, Stan A. Burgess, Alistair P. Curd, John A. Hammer, James R. Sellers, Philipp Kukura, Peter J. Knight

**Affiliations:** ^a^Physical and Theoretical Chemistry Laboratory, Department of Chemistry, University of Oxford, Oxford OX1 3QZ, United Kingdom; ^b^Laboratory of Single Molecule Biophysics, Biochemistry and Biophysics Center, National Heart, Lung, and Blood Institute, NIH, Bethesda, MD 20892; ^c^Laboratory of Molecular Physiology, Cell and Developmental Biology Center, National Heart, Lung, and Blood Institute, NIH, Bethesda, MD 20892; ^d^Astbury Centre for Structural Molecular Biology, and School of Molecular and Cellular Biology, Faculty of Biological Sciences, University of Leeds, Leeds LS2 9JT, United Kingdom; ^e^Cell and Developmental Biology Center, National Heart, Lung, and Blood Institute, NIH, Bethesda, MD 20892; ^f^The Kavli Institute for Nanoscience Discovery, Dorothy Crowfoot Hodgkin Building, University of Oxford, Oxford OX1 3QU, United Kingdom

**Keywords:** myosin-5, actin filament, cumulative angular disorder (CAD), interferometric scattering (iSCAT) microscopy, electron microscopy

## Abstract

Molecular motors employ chemical energy to generate unidirectional mechanical output against a track while navigating a chaotic cellular environment, potential disorder on the track, and against Brownian motion. Nevertheless, decades of nanometer-precise optical studies suggest that myosin-5a, one of the prototypical molecular motors, takes uniform steps spanning 13 subunits (36 nm) along its F-actin track. Here, we use high-resolution interferometric scattering microscopy to reveal that myosin takes strides spanning 22 to 34 actin subunits, despite walking straight along the helical actin filament. We show that cumulative angular disorder in F-actin accounts for the observed proportion of each stride length, akin to crossing a river on variably spaced stepping stones. Electron microscopy revealed the structure of the stepping molecule. Our results indicate that both motor and track are soft materials that can adapt to function in complex cellular conditions.

## Significance

Myosin-5 carries cargoes inside cells by walking along helical F-actin filaments. Myosin-5 avoids walking helically by taking long steps equal to F-actin’s helical repeat. Previous measurements of step length show a broader spread of values than expected from the spatial resolution of the technique. We used iSCAT and electron microscopies, which have subnanometer precision, and find that the broad peak is really a family of narrow peaks that correspond to steps spanning different numbers of subunits along F-actin. We show that disorder within F-actin is the reason myosin-5 varies its step length because the F-actin subunit best oriented for walking in a straight line changes from step to step. Thus F-actin disorder has important impacts on cellular functions.

**Fig. 1.**
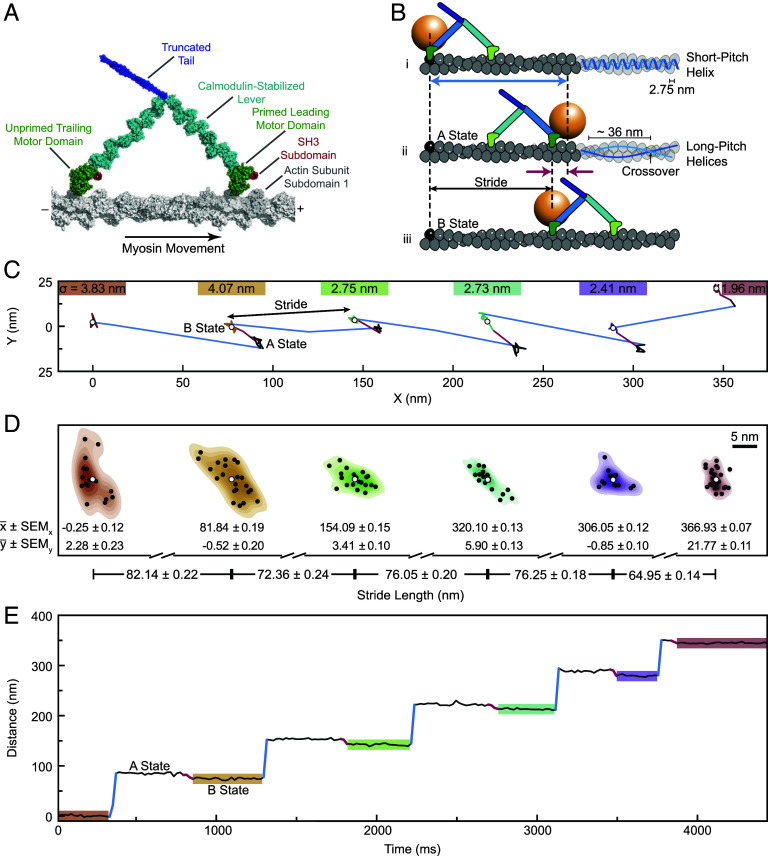
Trajectory along F-actin of a myosin-5a motor labeled with a gold bead. (*A*) Model of myosin-5 attached to F-actin (gray). The motor domains, bound mainly to the prominent subdomain 1 of actin subunits, are here 13 actin subunits apart. Note the different emergence point of the lever from unprimed and primed motor domains. The polarity of F-actin is indicated, with the barbed (+) end at right. (*B*) Schematic of two consecutive myosin steps, depicting first, the labeled head movement (blue arrow; B-state to A-state) and second, the AB transition of the bead (red arrows) accompanying the unlabeled head movement and allowing measurement of the labeled head stride (B-state to B-state). Also shown at *Right* is actin’s short-pitch helix (*Top* filament; the bar indicates separation of successive subunits along that helix) and two long-pitch helices (*Middle* filament; the bar indicates spacing of successive crossovers). (*C*) The XY trace of a typical run along F-actin of a gold-labeled myosin-5a molecule, highlighting labeled head strides (blue) and AB transitions (red). We report stride lengths as the Euclidean distance between the mean x and y values of consecutive B states. Localization precisions (σ), defined as the root sum of squares of x and y SDs of stationary states, marked above each B state. (*D*) The 2D kernel density estimates of bead positions highlighted in (*C*) with black points at each localization and white points at the mean values ($\bar{\;\text{x}}$, $\bar{\;\text{y}}$). Distances between the means demonstrate our high precision of stride length measurements. Uncertainty on mean particle position is SE on mean (SEM_x_, SEM_y_), propagated to the error on stride lengths. (*E*) Distance along the filament versus Time trace of the XY plot in (*C*), again, highlighting labeled head movements (blue) and AB transitions (red).

Myosin-5a is a cellular motor protein that carries cargoes along F-actin tracks (1–5). It has two motor domains connected to a coiled-coil tail by long levers (Fig. 1*A*), and globular domains at the tip of the tail that bind cargo. Each cycle of ATP hydrolysis by the motor fuels a cyclic swing of the lever from a backward-pointing, “primed” state to a forward-pointing, “unprimed” state, and this carries the detached motor forward to a new attachment site along F-actin (Fig. 1*B*) (1, 6–11). The molecule spends most of its kinetic cycle with both motors tightly bound to actin, so a single molecule can walk along actin without letting go (12–14).

The F-actin track is helical (15–18). Globular actin subunits form a single, left-handed, short-pitch helix having an axial separation of 2.75 nm and a more variable left-handed rotation of ∼-166°. In side-view, this gives an appearance of two coaxial, right-handed helices of subunits, staggered by a half subunit, that cross over one another every ∼36 nm (Fig. 1*B*). For myosin-5a to carry cargo in a straight line rather than winding around the helix, it must take steps equal to the helical repeat of actin, i.e., ∼36 nm (Fig. 1*B*). Indeed, previous studies that followed the molecule as it steps along F-actin have concluded that it takes steps of ∼36 nm (6, 9, 10, 13, 14), with a broad range of values (SDs ∼5.5 to 8 nm) ascribed to measurement uncertainties, despite the methods having nanometer precision (11, 14, 19–22). Here, we use interferometric scattering (iSCAT) microscopy with subnanometer precision (23–25) to reveal that this broad range actually comprises a series of discrete step lengths that were previously unresolved. We do this by attaching a 20 nm gold bead to one motor and following the strides of ∼72 nm that it takes as it moves from being the trailing motor to the new leading motor (Fig. 1*B*).

Myosin-5 walks at a constant azimuth along F-actin in the iSCAT assay (26), which seems to conflict with it taking steps of varying length on a regular helical track. However, evidence has accumulated that F-actin is an irregular helix, due to cumulative angular disorder (CAD) between neighboring subunits (27–29). The essence of CAD is that, within a single actin filament the left-handed rotation between successive subunits is not fixed but randomly variable, but the axial separation remains fixed. Thus, although a 26-subunit stride along F-actin is always 71.5 nm along the F-actin axis, the azimuthal change around the actin filament in taking the stride is variable. The implications of CAD for F-actin’s function as a track for myosin motors have been overlooked. However, by formalizing the link between CAD and the orientation of successive actin subunits along F-actin, we now show that the range and frequencies of step and stride lengths along F-actin can be explained by CAD causing variation in which actin subunit is best oriented for straight walking. Thus for myosin-5 to carry its cargo straight along F-actin, it has to vary its step length.

Early studies of myosin step length on F-actin by negative stain electron microscopy (nsEM) had shown a range of motor separations (6), but since this was not confirmed by measurements of actively stepping molecules, there was doubt that the nsEM results were reliable. Therefore, we have both repeated the nsEM approach under the solution conditions of the iSCAT assay and also used unstained cryogenic electron microscopy (cryoEM). Both studies show a range of step lengths, like in the iSCAT data, but with a smaller proportion of longer steps. The shorter average step length implies that myosin-5 walks left-handed around F-actin in solution, as has been previously demonstrated (30–32). The EM data also show the overall structure of the stepping molecule, and reinforce the earlier conclusion (6, 33) that in the doubly attached molecule, the leading motor is held in a near-primed structure by tethering through the levers to the trailing motor.

## Results

### iSCAT Resolves Varying Stride Lengths of Myosin-5a.

Molecules of a myosin-5a construct labeled on the N terminus of one motor with a 20 nm gold bead were tracked by iSCAT microscopy at 100 Hz as they walked along actin filaments in the presence of 10 μM ATP. For each molecule, the x, y position of the bead was determined with subnanometer precision (0.9 nm localization precision) (26) in each frame, allowing detection of the stride of the labeled motor and also the AB transition of the bead that accompanies the stride of the unlabeled motor (26) (Fig. 1 *C* and *E*). The difference between mean positions of the bead in successive B-states yields the length of each stride taken by the labeled head (Fig. 1*B*–*D*).

**Fig. 2.**
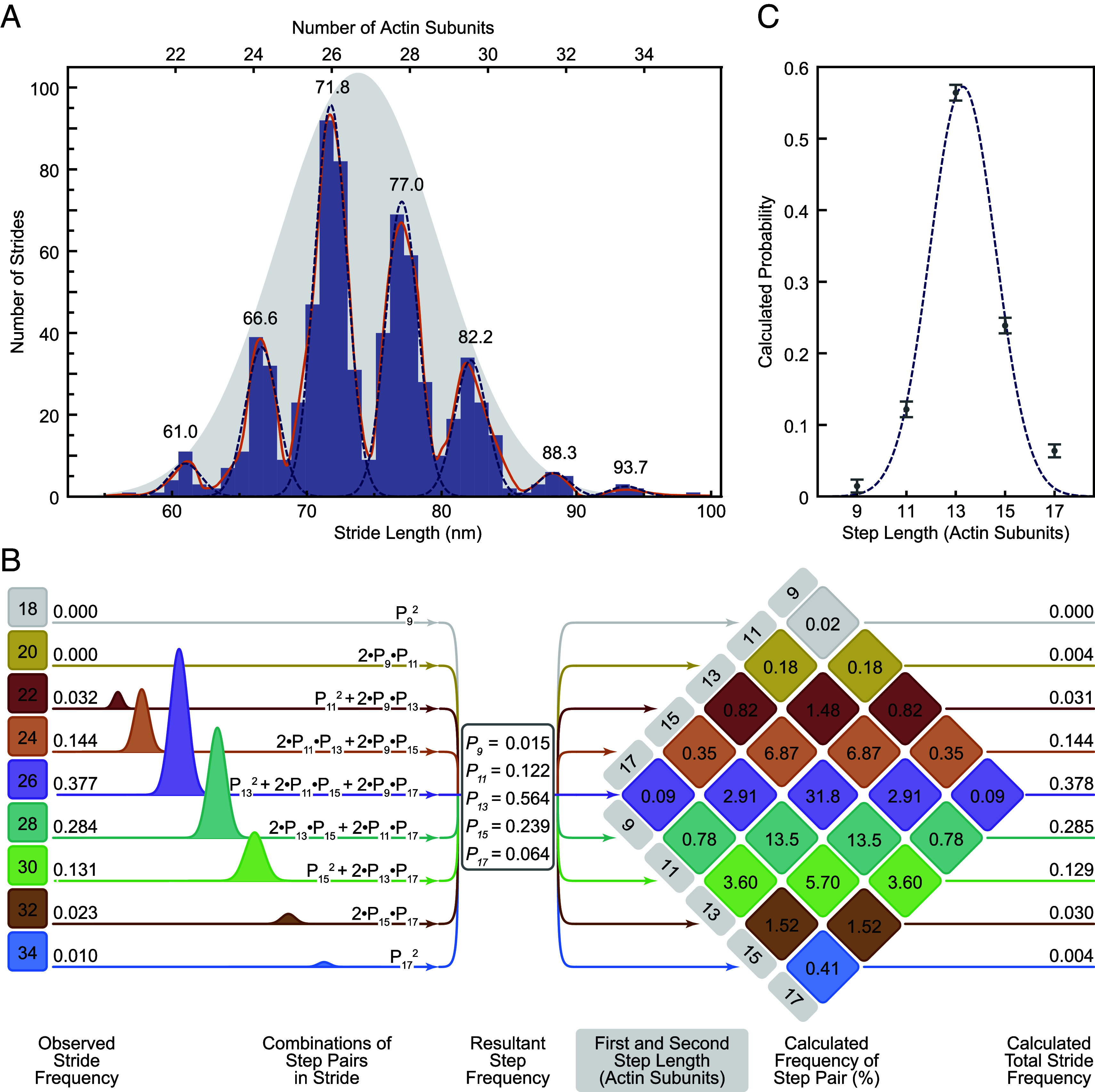
Analysis of strides taken by myosin-5a on F-actin. (*A*) Histogram of measured myosin-5 strides (*N* = 96 molecules, 725 strides). A Gaussian kernel density estimate, with locally adaptive bandwidth, is shown (solid orange line), as well as seven individual Gaussians (dashed lines). Mean stride length (nm) of each Gaussian is labeled above the fits, and calculated number of actin subunits traversed marked. The single Gaussian that expresses the overall mean and SD of the dataset is shown (gray shaded), rescaled to show fit to relative frequencies of the strides. (*B*) A schematic depicting the set of nine equations used to calculate the probability of a given step length (P_x_, where x = 9, 11, 13, 15, 17 actin subunits) from the areas of the Gaussian fits to the stride peaks listed on the *Left* side. *Center* panel lists the calculated step length probabilities. On the *Right* is the multiplication diamond using the calculated probabilities of each step length to determine the percentage frequency of a stride resulting from each combination of step lengths. Combinations of step lengths that result in the same stride length are of the same color. The total predicted frequencies of each stride length are listed on the *Right* for comparison with the observed frequencies listed on the *Left*. (*C*) Step probabilities with fitted Gaussian.

The histogram of measured stride lengths is strikingly multimodal, with very little overlap between adjacent peaks (Fig. 2*A*). This demonstrates unequivocally that myosin-5a takes strides of varying but quantized length and that iSCAT microscopy has the spatial precision to resolve these. The separation of adjacent peaks of 7 Gaussians fitted to the binned data is 5.45 ± 0.40 nm (mean ± SD), which corresponds to a separation of two actin subunits along the filament (5.5 nm), that is, it corresponds to neighboring subunits along one of the two long-pitch strands (Fig. 1*B*). The major peak at 71.8 nm corresponds to a distance spanning 26 actin subunits (expected value, 71.5 nm). This is the stride length expected from two canonical 13-subunit steps. The other peaks therefore correspond to strides that land on subunits nearby to the 26th along the same long-pitch actin strand. The family of peaks thus correspond to strides spanning 22, 24, 26, 28, 30, 32, and 34 actin subunits. Using the average of all the peaks, weighted by their normalized area, the spacing of subunits along F-actin is 2.758 ± 0.016 nm. Since the myosin motor binds stereo-specifically to actin (34), the width of each peak likely arises from imprecision of measurement. This is expected to be independent of stride length, and indeed, an excellent fit to the data is obtained using a single width parameter (SD = 1.195 nm) for all seven peaks (Fig. 2*A*).

Using iSCAT microscopy, it is therefore possible to describe any individual processive run by the precise numbers of actin subunits traversed in each of the strides. For example, the run shown in Fig. 1*C*–*E* can be described as 30, 26, 28, 28, 24.

The data indicate that all molecules behaved similarly. Thus, comparison of the trajectories of the 96 molecules (*SI Appendix*, Fig. S2*A*) shows that all molecules take a range of stride lengths, rather than some being inherently short-striders and others, long-striders. Also, the distribution of stride lengths is independent of the number of strides taken in a processive run (*SI Appendix*, Fig. S2*B*). Furthermore, a $\chi^{2}$ analysis showed that the length of each stride was independent of the length of the preceding stride. These are all features to be expected if successive strides are independent events.

The dataset of strides has an overall mean of 73.7 nm and SD of 5.66 nm. These overall values are close to those obtained in earlier studies using other methods that did not resolve the component peaks (11, 19, 21, 22). This indicates that the flexibility in stepping that we observe by iSCAT was also present in all those studies. This mean and SD can be represented by a Gaussian distribution and scaling its amplitude shows that it is a good fit to the relative amplitudes of the component peaks (Fig. 2*A*). This indicates that the underlying cause of variable stride length has a Gaussian character. We investigate this further below.

It is important to note that the overall mean value of stride length falls between our observed peaks, and thus does not correspond to a stride length that is ever taken. This is because binding sites for motors on F-actin are not separated by this mean value.

By using high-resolution iSCAT microscopy, we have thus revealed that molecules of myosin-5a take strides of variable length during a single processive run.

### Myosin-5a Step Frequencies Can Be Estimated from the Stride Frequencies.

Quantitative analysis of the relative frequencies of each stride length (Fig. 2*B*) reveals further insights into the stepping behavior of myosin-5a. Each stride length is the sum of a forward step by the unlabeled motor followed by the labeled motor. For example, a 24-subunit stride can result from four different combinations {1st step subunits, 2nd step subunits}: {11, 13}, {13, 11}, {9, 15}, and {15, 9} actin subunits (Fig. 2*B*). Using the measured relative frequencies of the seven stride lengths (areas under the fitted peaks; Fig. 2 *A* and *B*), we found the least squares best-fit solution to the set of simultaneous equations containing the probabilities of the five underlying step lengths (motors spanning 9, 11, 13, 15, and 17 subunits) (Fig. 2*B*, *Left* panel). The fit was robust and excellent, with only small differences between the observed frequencies and those backcalculated from the fitted values of step probabilities (Fig. 2*B*, *Right* panel). The values of step probabilities versus step length are well fitted by a single Gaussian of mean 13.3 with SD 1.32 actin subunits (with associated fitting errors, 0.12 and 0.10 actin subunits, respectively) (Fig. 2*C*). These properties are strong evidence that the walking process is the result of a random and independent selection of step lengths governed by an underlying Gaussian probability distribution.

The range of step sizes that is implicit in the multiple stride lengths shows that the canonical 13-subunit step is not as abundant as previously thought. Only 56% of all steps are 13-subunits long (Fig. 2*B*, *Middle* panel, and Table 1). From the calculated relative abundance of the various step lengths (Fig. 2*B*), the average step length is 13.469 actin subunits (= 37.04 nm) (Table 1). Likewise, only 38% of the strides are 26-subunits, and this includes combinations of 11+15 subunit steps in addition to two 13-subunit steps. Only 32% of all strides are a combination of two consecutive 13-subunit steps (Fig. 2*B*, *Right* panel).

**Table 1.** Calculated probabilities of each step length from iSCAT, cryoEM, and nsEM imaging

Step length	Step probability		
(actin subunits (asu))	iSCAT	cryoEM	nsEM
9	0.014		0.025
11	0.122	0.106	0.081
13	0.564	0.770	0.754
15	0.239	0.125	0.127
17	0.064		0.013
Mean Step (asu)	13.469	13.038	13.044
SD (asu)	1.585	0.966	1.196
			
Mean Step (nm)	37.039	35.855	35.871
SD (nm)	4.357	2.650	3.289

The mean step length and SD in step lengths are also tabulated.

This analysis to determine step frequencies shows that myosin-5a is a more flexible stepper than has been widely recognized, and that the widely stated 36 nm step accounts for only about half of the steps taken.

### Structure of Myosin-5a on Actin Using cryoEM.

We used cryoEM to examine the structure of full-length myosin-5a during stepping on actin, to test the earlier conclusions from nsEM. In practice, it proved difficult to get these samples into the holes of the support film, but a small dataset has been obtained that delineates the structures present.

In the presence of ATP, at low calcium concentration and with no cargo adaptor molecules, myosin-5a is mainly compactly folded (35–38). These folded molecules are autoinhibited and bind only weakly to actin (38). However a small fraction of molecules is found to be active and to move processively along actin (38), so these should be present in samples flash frozen and examined by cryoEM. 55% of the myosin molecules observed were in the extended conformation and attached by both heads to the same actin filament (*SI Appendix*, Fig. S3). This abundance of myosin-5a molecules attached to actin is not surprising because we used a high ratio of myosin to actin (1 molecule per 2.5 actin subunits). Counts of myosin molecules per μm actin suggest that less than 10% of the added myosins were attached, which is consistent with the typical ∼10-fold regulation of actin-activated ATPase activity in full-length myosin-5a preparations (36). The relative scarcity of detached molecules may arise from their adsorption to the carbon film.

**Fig. 3.**
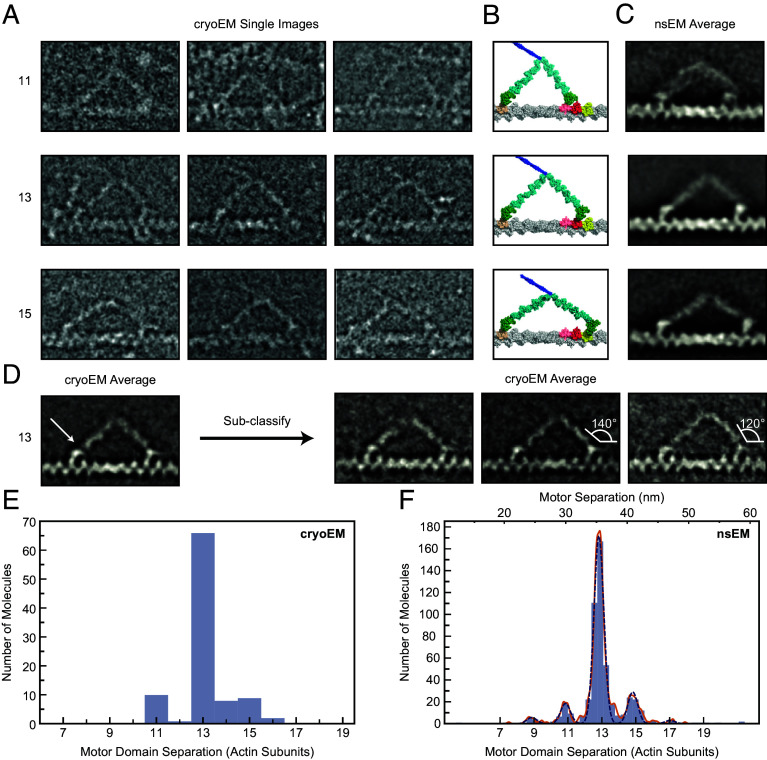
CryoEM and nsEM of myosin-5a walking on F-actin. (*A*) Gallery of single cryoEM images depicting myosin-5 with step lengths 11, 13, or 15 actin subunits. Contrast inverted (protein white). (*B*) Atomic models corresponding to (*A*), constructed according to Vale and Milligan (39), assuming a 13/6 F-actin helix. Actin subunits 11, 13, or 15 subunits away from the trailing motor domain are colored pink, red, and yellow, respectively. (*C*) Averaged nsEM images of myosin-5 with step lengths 11, 13, or 15 actin subunits. For the step length 11 subunits, the leading motor has an unprimed conformation, unlike for 13 and 15 subunit steps. (*D*) *Left* panel: averaged cryoEM image of myosin-5 with step length 13 actin subunits. Arrow indicates the low density between motor and lever of the trail head. *Right* panels: result of classification into three subclasses using features in the leading lever. The right subclass has a convex leading lever shape, but the leading motor domain still has a near-primed conformation. White diagrams highlight the smaller angle between the actin axis and the emerging lever for the convex lever than the straight lever. (*A*–*D*) Actin barbed (+) end and myosin-5a leading head are on the right in all panels. Panels (*A*–*D*) are scaled to match the panels in (*B*), in which 13 actin subunits span 35.75 nm. (*E*) Separation between motor domains from cryoEM data. The apparently even-numbered motor domain separations arise from the tails of the error distribution associated with measurement. No even-numbered separations are observed in the images. (*F*) Separation between motor domains from nsEM data. A Gaussian kernel density estimate, with locally adaptive bandwidth, is shown (solid orange line), as well as five individual Gaussians (dashed blue lines). The number of actin subunits is shown along the *Bottom*, and the corresponding separation distance (nm) along the *Top*.

In raw cryoEM images, the motor domains, levers and the first, coiled-coil segment of the tail are frequently identifiable (Fig. 3*A*). The two heads of doubly attached molecules typically show asymmetry recalling the appearance seen in negative stain (6), with the two levers pointing in opposite directions. A global average of doubly attached molecules with motor domains 13 actin subunits apart improves clarity (Fig. 3*D*). Both motor domains are attached on the leading side of subdomain 1 of actin (compare with the atomic model, Fig. 1*A*), with the N-terminal SH3-fold subdomain extending as far forward as the axial position of subdomain 1 of the next actin subunit, as in models of primed and unprimed heads on actin (Fig. 1*A*) (40). There is lower density between the motor and lever in the trail head than in the lead head (arrow, Fig. 3*D*), again in accord with the expectations from molecular models. The levers are visible throughout their length and unite at the head–tail junction without a gap, showing that the two polypeptide chains of the proximal coiled coil tail are not stretched apart by the stress within the doubly attached molecule. All these features recall those seen using nsEM (6, 33), and which can also be seen in averages from a new nsEM dataset (Fig. 3*C*). An exact correspondence between the cryoEM and nsEM averages should not be expected as they are fundamentally different imaging modalities, and there is flattening during drying in nsEM (41) that is avoided in cryoEM.

In the trailing head, the lever emerges from the leading side of the motor, near the SH3 domain, as expected for an unprimed head. The angle between the trailing lever and F-actin axis in the global average where the two motor domains are 13 subunits apart is 40°, as previously found by nsEM at rate-limiting, low ATP concentration (39° to 49°) (7) in which the trailing heads were largely devoid of ADP. This indicates that release of ADP from the trailing head has little effect on the overall geometry of the doubly attached myosin-5a, in contrast to the small lever swing found for single myosin-5a heads attached to actin when ADP is released (13, 34).

The leading heads show an abrupt angle between motor and lever and the lever emerges from the trailing side of the motor, indicating that the converter is in a near-primed position, despite the motor having released phosphate (7, 42) (Fig. 3*D*). Classification of the leading lever region shows variation in shape, with some levers convexly curved away from the actin filament and emerging from the motor at ∼120°, rather than straight and emerging from the motor at ∼140° (Fig. 3*D*, *Right* panels). The curved levers still emerge from the trailing side of the motor domain, not the leading side. Thus in these cryoEM data, the leading motor domains are in a near-primed conformation, not unprimed.

The cryoEM data demonstrate that the observations made previously using nsEM (6, 7) are not artifacts of staining and drying. In particular, the two motor domains of the doubly attached molecule are in different structural states despite being in the same biochemical state. This may underlie their different rates of ADP release (42, 43).

### Myosin-5a Step Size Distributions from Electron Microscopy.

Myosin-5a molecules with both heads attached on the actin filaments were identified and the separation between the two motor domains of the myosin molecule was measured by an unbiased method, whereby the positions of both motors were determined independently following alignment and classification. For this analysis, it was not necessary to determine the polarity of the F-actin filament or to identify which of the two motors was the lead or trail head. In the cryoEM dataset, motors are found to be separated by 11, 13 and 15 actin subunits (Fig. 3*A*). 77% of molecules have motors attached 13-subunits apart, 10% have motors 11-subunits apart and 13% are 15-subunits apart (Fig. 3*E*). The mean separation is 13.038 actin subunits (= 35.86 nm) (Table 1).

A new dataset was also obtained using nsEM of the same myosin-5a construct as used for iSCAT and walking along phalloidin-stabilized F-actin under the same conditions as the iSCAT assay, including 10 μM ATP rather than the 1 μM ATP used in earlier studies (6, 33). This higher ATP concentration should produce a higher fraction of doubly attached molecules that have ADP in the trailing motor domain (≈50% at 10 μM ATP cf. ≈10% at 1 μM ATP). Analysis shows 9-, 11-, 13-, 15-, and 17-subunit separations (Fig. 3 *C* and *F*). Unlike in longer steps, the global image average of molecules stepping 11 subunits shows the leading head lever emerging from the leading side of the motor domain. This indicates that the smaller separation of motor domains allows the leading motor domain converter to move to the unprimed position, as found previously (33) with concomitant distortion in the proximal lever. Like in the cryoEM data, 13-subunit steps are strongly favored (75%), with a mean step length of 13.044 actin subunits (35.87 nm) (Table 1). Suppression of CAD may account for the narrower distribution of step length in both sets of EM data: in cryoEM by physical forces in the thin aqueous film prior to freezing (44, 45), and in nsEM by phalloidin strengthening interactions between adjacent actin subunits (46). Phalloidin stabilization, which by increasing the rotation per actin subunit (46) moves the 13th subunit further from straight ahead, and brings the 15th subunit closer to straight ahead, has little effect on the step-length probabilities. The EM data complement the iSCAT data by showing the step lengths directly whereas the iSCAT data show strides, which each contain a pair of steps. Both EM methods show a reduced proportion of 15-subunit steps compared with iSCAT.

### How Can a Straight-Walking Myosin Molecule Be Taking Variable Length Strides?.

**Fig. 4.**
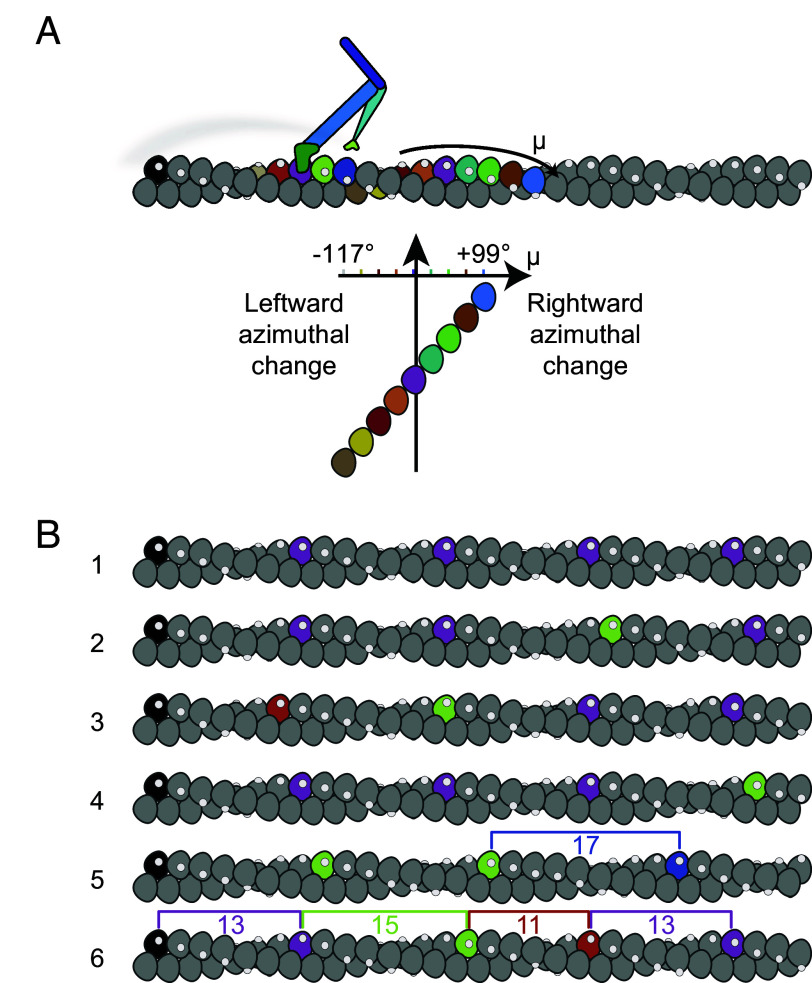
Implications of variable step length if F-actin has fixed or disordered helical parameters. (*A*) Schematic detailing how varying step and stride lengths would rotate myosin-5a trajectory around the actin filament given a fixed actin rotation per subunit, ϕ = −166.39°. Motor binding site on actin subunit depicted as white spot. A myosin-5a molecule is schematically shown at a stage where one motor (pale green) has detached from the black actin subunit and the attached motor (dark green) has undergone its power stroke. The detached motor is transiently dwelling off-axis, behind the plane of the figure and will bind to the right of the attached motor. Stride color scheme matches Fig. 2. (*B*) F-actin with variable CAD. Filament 1 has fixed subunit rotation −166.39° and axial translation 2.75 nm. Filaments 2 to 6 were built incrementally from left to right by adding subunits with an addition to the rotation per subunit randomly drawn from a Gaussian distribution with mean 0° and SD 5.28°. Actin subunits that lie closest to the same azimuth as the zeroth (black) subunit are highlighted with the step colors used in (*A*), to show expected myosin step lengths, indicated by brackets on filaments 5 and 6.

If F-actin was a perfectly regular helix, i.e., had a fixed rotation per subunit, our observation of randomly variable step length would imply that the myosin-5a molecule walks drunkenly along actin, moving left and right around the filament as well as along it (Fig. 4*A*). However, in our iSCAT assay, myosin-5a walks straight along actin, staying perpendicular to the plane of the microscope coverslip to which the actin is bound (as described previously (26); see also *SI Appendix*, *Text*). A possible solution to this paradox is that CAD in F-actin could force myosin-5a to vary its step length to continue walking straight. This would be equivalent to someone walking across a river using stepping stones that are spaced at variable separations along a regular lattice (Fig. 4*B*). Thus, the relative frequencies of stride lengths would reflect the characteristics of CAD in the F-actin used in the iSCAT assay. We have therefore tested whether CAD in F-actin could be of sufficient magnitude to account for the observed striding behavior. In doing this we have enlarged upon two methods for assessing CAD from images of F-actin (28), using recently published cryoEM images of F-actin (17), and show that CAD is indeed present (*SI Appendix*, *Text* and Figs. S4–S7).

### Can CAD in F-Actin Account for the Varying Stride of Myo-sin-5a?.

The implication of CAD for myosin-5a walking is as follows. The mean azimuth (μ_*n*_) of the *n*th actin subunit, in degrees, relative to that of a starting subunit having an azimuth ($\mu_{0}$) of 0° is given by

[1] $$\begin{array}{c} \mu_{n}=n\phi \;\;\text{modulo}\;360, \end{array}$$

where ϕ is the mean rotation per subunit in the filament. The SD ($\sigma_{n}$) of that azimuth depends on the RMSD disorder value, d, and the number of subunits through:

[2] $$\begin{array}{c} \sigma_{n}=d\sqrt{n}. \end{array}$$

**Fig. 5.**
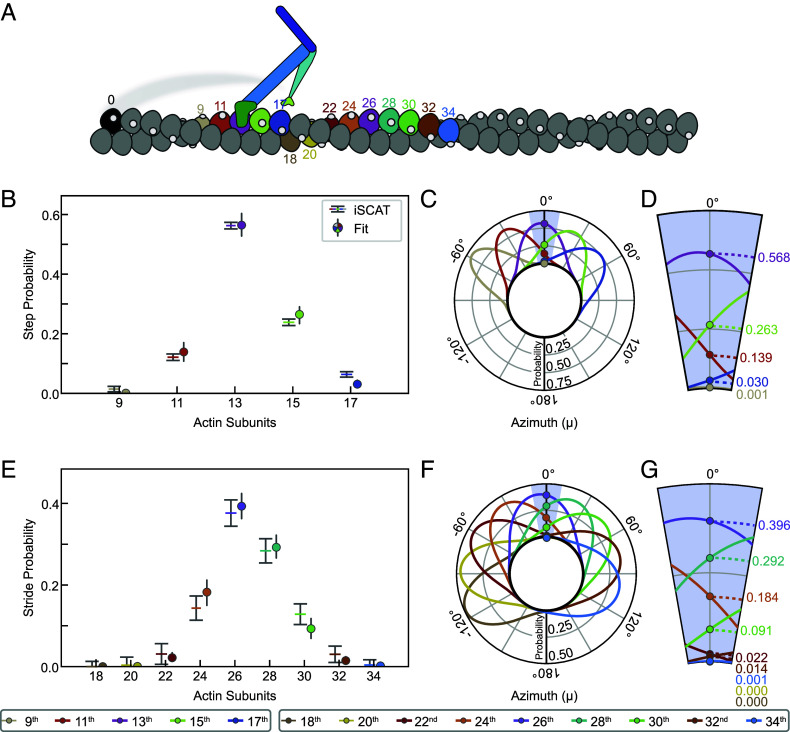
Comparison of F-actin cumulative angular disorder with myosin-5a step and stride length distributions. (*A*) Schematic numbering the actin subunits involved in steps and strides. Color scheme matches Fig. 4. (*B*) Calculated probabilities of each step length from iSCAT data, compared with probabilities from the angular disorder $\theta^{{}^{\circ}}$ azimuth fit. (*C*) Radial plot, i.e., as if looking along the F-actin axis towards the barbed end, showing the azimuthal probability distributions of the *n*th subunit away from the initial (i.e., the black) subunit along F-actin with the subunit rotation and CAD values ($\phi =-166.39\pm 5.28^{{}^{\circ}}$) obtained from least squares fitting to the iSCAT data. Probability densities have been normalized such that the sum of probabilities at $\mu =\theta^{{}^{\circ}}$ is 1. (*D*) Expanded $\pm 10^{{}^{\circ}}$ region showing probabilities of the *n*th subunit lying at $\mu =\theta^{{}^{\circ}}$. (*E*, *F* and *G*) Comparable to (*B*, *C* and *D*, respectively), but refer to strides rather than steps.

Thus, for any pair of values of ϕ and d, one can estimate the probabilities of the 9th, 11th, 13th, 15th, and 17th subunits being closest to an azimuth of 0° and thus the preferred target for straight walking (Fig. 5*A*). It is important to appreciate that on the rare occasions when the 17th subunit is optimally positioned, the 13th will be at μ$\approx -55^{{}^{\circ}}$, requiring a significant reach around the actin. We now apply these principles to test whether CAD can account for the relative frequencies of each step and stride length.

If the variable step lengths in the iSCAT data were solely a response to CAD in actin, then our estimates of the relative frequencies of 9, 11, 13, 15 and 17-subunit steps should closely match the relative probabilities of those subunits being correctly positioned for straight walking due to CAD. We therefore used our five stepping frequencies to constrain least squares best-fit estimates of ϕ and d in the set of five pairs of equations for μ_n_ and σ_n_ to test whether the resulting values agree with current estimates. A robust fit was found, yielding $\phi =-166.39^{{}^{\circ}}\pm 0.11^{{}^{\circ}}$ and $d=5.28^{{}^{\circ}}\pm 0.26^{{}^{\circ}}$. Both these values are close to previous estimates, and there is an excellent match between the measured and fitted probabilities of the five step lengths (Fig. 5*B*). Radial plots of the probability distribution illustrate the range of azimuth that each target actin subunit occupies, as viewed along the actin filament axis (Fig. 5*C*). The enlarged segment around $\mu =0^{{}^{\circ}}$ shows the azimuthal dependence of probability for straight walking (Fig. 5*D*). For the 17th subunit, the shallow slope of this dependence accounts for the relatively narrow error limits. We conclude that the presence of CAD in F-actin is sufficient to quantitatively account for the variable step lengths taken by myosin-5a.

If CAD is indeed the origin of variable step lengths in the iSCAT assay, then it should also account for the variable stride lengths. The ϕ and d parameters of the actin filament derived from stepping probabilities were therefore used to predict the relative probabilities of the 22nd to 34th subunit positions that are the target sites for the striding motor. These also give a very good match with the observed frequencies (Fig. 5*E*). The radial plots of probability distribution for strides (Fig. 5 *F* and *G*) are noticeably broader than those for the steps, demonstrating the progressive reduction of the correlation with the starting azimuth that is a feature of cumulative (liquid-like) disorder. This good fit to the striding data indicates that CAD in actin does indeed account fully for the spread and relative frequencies of strides observed in the iSCAT data.

Our analysis demonstrates that typical amounts of CAD in F-actin are sufficient to dictate a widely varying stepping pattern for a myosin-5a molecule walking straight.

### Step Lengths Found by EM Indicate Myosin-5a Walks Left-Handed around Free F-actin.

The EM data give a complementary view of myosin-5a stepping to that of iSCAT, in that the myosin is walking along F-actin that is free in solution, rather than apposed to a surface, at the point of EM grid preparation. This is similar to previous data on myosin-5 twirling around single actin filaments suspended between supports, and these showed a net left-handed rotation during stepping (30–32). For the cryoEM dataset, longer steps are rarer than in iSCAT, indicating that myosin-5a prefers to take 13-subunit steps even when CAD means that it must move left-handed around the F-actin axis to do so. The average step length 13.038 actin subunits) is therefore shorter than the average iSCAT step length (13.469 subunits). As a result, using the value of −166.39° for mean actin rotation per subunit, derived above from the iSCAT data, together with the proportion of each step length in the data, yields an average azimuthal movement that is left-handed of −2.55° per step. This value implies that on average, myosin-5a would take 141 steps to complete a left-handed rotation around the filament, while moving 5.1 μm along it. The nsEM dataset is very similar, yielding an average step length of 13.044 subunits. Since the nsEM data were obtained using phalloidin-stabilized F-actin that has a rotation per subunit of −167° (46), the mean rotation per step is −10.43°, so a complete left-handed rotation would require only 36 steps covering a distance of 1.3 μm.

### Does Step Length Influence Myosin-5a ATPase Kinetics?.

**Fig. 6.**
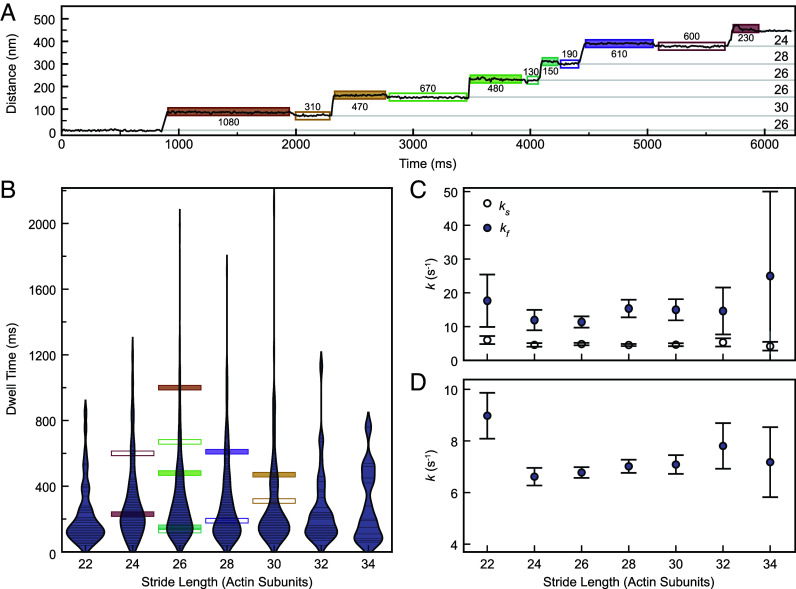
Analysis of impact of stride length on dwell times of *A* and *B* states. (*A*) Distance vs. Time trace of a typical gold-labeled myosin-5a motor domain demonstrating variability in dwell times of the labeled motor domain. Dwell times (ms) marked below *A* states (filled boxes) and above B states (empty boxes). (*B*) Measured dwell time of *A* states after stride and *B* states before stride as a function of stride length. Each observed dwell is shown as a horizontal line, and the dwell times measured from the trace in panel (*A*) are highlighted using the same box styles. Violin plot envelopes are kernel density estimates of the distributions with bandwidth of 150 ms. (*C*) Fitted variable rate constants, $k_{s}$ and $k_{f}$, as a function of stride length. (*D*) Shared fit variable rate constant, k, as a function of stride length. Error bars in (*C*) and (*D*) denote the SE of the fit parameters obtained from the observed Fisher information matrix.

When myosin-5a takes a long step, the levers necessarily lie at a smaller angle to the actin filament than when it takes a short step, as is indeed seen by EM (Fig. 3). Since release of ADP from the head moves the lever to a smaller angle to actin (13, 47) it might be expected that when the motors are separated by more actin subunits, the rate of ADP release from the trail head would be accelerated, resulting in a shorter dwell time, than would be observed with shorter separations. Conversely, short steps suggest a larger lever angle, slower ADP release, and longer dwell time. Because iSCAT resolves varying stride lengths, we have been able to test this idea. There is the complication that a stride may comprise a short step plus a long step in either order, but the shortest strides will comprise only short steps, or longest strides only long steps. The two dwell times that refer to a given stride are those of the B state that precedes it and the A state that follows it (Fig. 1*E* and Fig. 6*A*). Therefore, we have analyzed these dwell times for each stride length.

We found that there was no significant difference between the dwell times for the A states compared to those of the B states, regardless of stride length, in agreement with our earlier study (26), and thus confirming that the gold bead did not affect stepping kinetics. Violin plots of combined A-state and B-state data were similar across all stride lengths, with the most common stride lengths showing the greatest range of values, as expected (Fig. 6*B*). Consequently, there was meager evidence of dependence of rate constant on stride length (Fig. 6 *C* and *D*, and *SI Appendix*, Fig. S8), with rate constants for the two fitting models of $k_{s}\approx 4.5\;\;{\text{s}}^{-1}$ and $k_{f}\approx 12\;\;{\text{s}}^{-1}$ (Fig. 6*C* and *SI Appendix*, S7*A*) or $k\approx 7\;s^{-1}$ (Fig. 6*D* and *SI Appendix*, S8*B*) and no significant difference between the models as a fit to the data. We conclude that the walking rate, and thus ATPase kinetics, of myosin-5a is not greatly dependent on step length.

## Discussion

Using iSCAT microscopy, we have succeeded in resolving a family of stride lengths taken along F-actin by myosin-5a that has one head labeled with a gold nanoparticle. The overall mean and SD of the dataset are similar to those of earlier studies that did not resolve the component peaks, indicating that these variable strides are a constant feature of myosin-5a stepping.

**Fig. 7.**
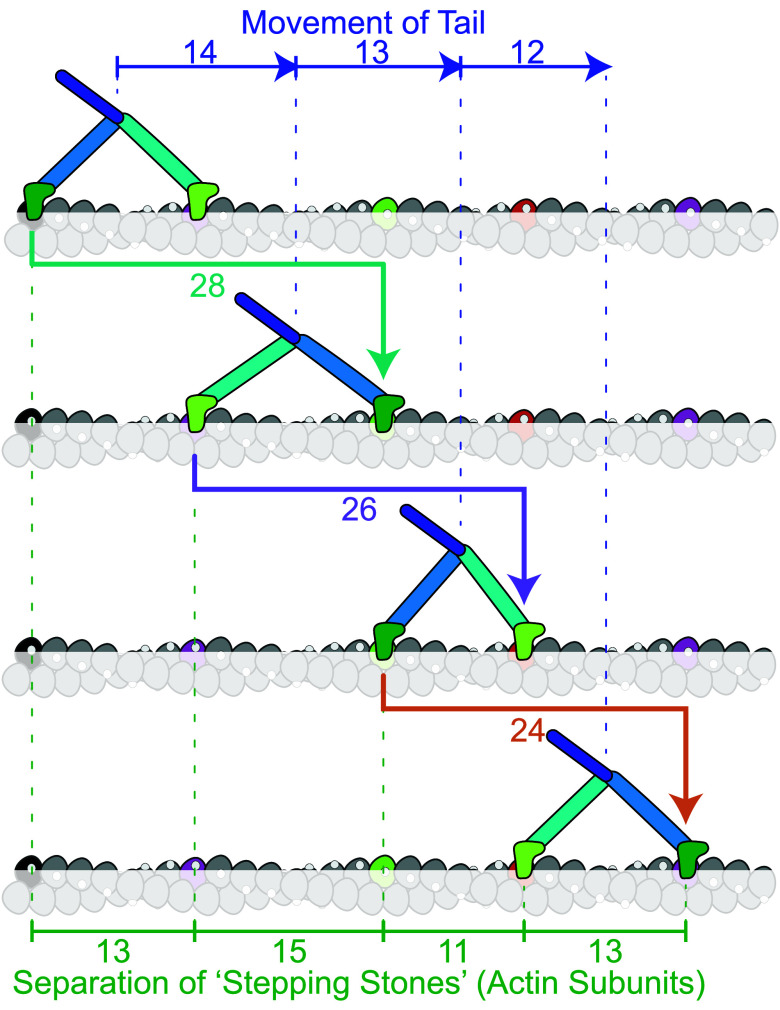
Variable striding of myosin-5 along irregularly spaced F-actin “stepping stones.” A model actin filament (taken from Fig. 4*B* filament 6) that incorporates CAD, happens to have subunits at similar azimuths to the starting (black) subunit at subunit separations of 13 (purple), 15 (green), 11 (red) and 13 (purple). Other subunits are less favored for stepping, as if submerged (grayed out). The two heads (dark and light colored) of the straight-walking myosin-5 molecule alternate to take strides that therefore traverse 28, 26, and 24 subunits (77, 71.5, and 66 nm, respectively). Notice that the forward movements of the tail, and thus any attached cargo, do not match the separations of the stepping stones; instead, they are the average of successive pairs of separations.

Why were these variable stride lengths not resolved in previous studies? One seemingly trivial reason is that because the strides differ in length by one actin subunit distance (5.5 nm) it is necessary to aggregate the data into small enough bins (≤1.8 nm) to resolve that spacing, yet this has generally not been done. A second reason is that the method used to determine the start and end positions of each stride can add sufficient error that the peaks overlap. A third reason is that determining stride lengths by the separation between dwell-time plateaus of distance-time plots (e.g., Fig. 1*E*) is subject to error arising from the systematic underestimation of bead movements orthogonal to the direction of movement. Thus, for the iSCAT data, a switch from measuring lengths from distance-time traces (26) to measuring them from the x, y coordinates proved critical to resolving the strides. A further challenge arises where the center of mass of the molecule is being monitored, such as if both heads are labeled or in optical trap studies. Only if successive motor separations along actin are all the same is the movement of the molecule equal to the motor separation. If motor separations vary, as we have shown they do, then in making the transition between them, the center of mass moves by the average of the two separations. For example, if a motor separation of 13 subunits is followed by one of 15 subunits, the center of mass moves 14 subunits (Fig. 7). This means that the movement of the center of mass is reporting half the length of the strides rather than the sizes of the steps taken by the motors. It also means that it is more demanding to resolve the subpeaks because they are only 2.75 nm apart.

### Misconception of the Myosin-5a Average Step Length Explained.

In contrast to common statements that myosin-5a walks by taking 36 nm steps along actin, we find that only about half the steps span the canonical 13 subunits (35.75 nm). Almost a quarter span 15 subunits with progressively smaller proportions spanning 11, 17 and 9 subunits. Because of this diversity of step lengths, neither the average step nor average stride length corresponds to an actual movement, as these average values fall between the separation of actin-binding sites. These results from the iSCAT assay pertain also to previous assays that used F-actin attached to a coverslip or suspended between a pair of beads in the optical trap. This is because in each case only a limited azimuth of F-actin is available for the myosin to walk along. The presence of a family of unresolved steps or strides in the previous data explains why the breadth of the observed distribution was broader than would have been expected from the resolution of the methods used.

### Cumulative Angular Disorder of Actin Filaments Accounts for Myosin-5A Step and Stride Frequency.

We show that CAD exists within F-actin specimens prepared for cryoEM by modern methods. Remarkably, CAD is of sufficient magnitude to account for the relative frequencies of the step and stride lengths found in the iSCAT data for myosin walking along a single azimuth of the actin filament. This nicely resolves the paradox of how myosin-5a could take steps of varying length while still walking straight on the helical F-actin. Recent high-resolution cryoEM structures of F-actin (17, 18, 48–50) disregard this feature of F-actin structure, instead emphasizing a precise (average) value for subunit rotation (close to −166.5° per subunit). However to obtain high resolution, these studies use thin ice, which has been shown to suppress CAD by shear and compressional forces (44, 45). Thus, high-resolution cryoEM can lead to misleading conclusions by suppressing inherent structural flexibility. High-resolution studies also restrict the reconstructed volume to 3 to 8 actin subunits (17, 51) which further reduces the impact of disorder. When a longer segment of filament is used, resolution falls (17), as is expected from CAD.

Our study has revealed the biological significance of CAD in influencing the behavior of myosin motors. Although CAD has been well-characterized for F-actin purified from skeletal muscle (27, 28), there are no data on CAD in cytoskeletal actin isoforms (52), and also no data for the influence of cytoskeletal tropomyosin or for CAD in actin filaments bundled by cross-linking proteins such as fascin or fimbrin. Although for some of these complexes, there are data on filament flexibility, there is no established causal linkage between the magnitude of CAD and flexibility. Our study therefore indicates a need for further research to characterize CAD in the cellular environment and to understand the roles of CAD in determining cellular behavior. Understanding the temperature dependence of CAD magnitude and dynamics would also be beneficial for understanding the importance of CAD in F-actin under physiological conditions for warm- and cold-blooded animals.

### Myosin-5a Walks Left-Handed around F-actin.

Previous studies have directly shown that myosin-5a molecules can walk left-handed around a suspended actin filament that has been stabilized by phalloidin (30–32). Phalloidin increases average actin subunit rotation to −167° (46). This moves the 13th subunit to an average azimuth 11° left of straight ahead and correspondingly the 15th subunit moves to be only 15° to the right. Thus, walking straight on such filaments requires a high proportion of 15 subunit steps. For myosin taking mainly 13 subunit steps, as we observe by both cryoEM and nsEM, the molecule would twirl left-handed around the filament more strongly than for phalloidin-free F-actin. This may account for the shorter pitch of twirling (∼2 to 3 μm) (30–32), which is comparable to our prediction of 1.3 μm for phalloidin F-actin and shorter than our prediction of 5.1 μm for phalloidin-free F-actin. This can be compared to an average run length of ∼1.3 μm (53–55), from previous studies. The earlier conclusion that left-handed twirling along phalloidin-stabilized F-actin implied that myosin took 11 and 13 subunit steps (30) is incorrect, because it assumed −166.154° rotation per subunit for F-actin (i.e., 13/6 helical symmetry) rather than −167°.

### Structure of the Walking Myosin-5A Molecule.

In studying myosin-5a walking on F-actin by cryoEM, we found it difficult to get the walking molecules into the holes of the EM support film. Nevertheless, our small dataset clearly shows that the converter of the leading motor domain is held in a stressed, near-primed position through tethering to the trail head. Thus, the flash-frozen, unstained walking molecule appears similar to our previous studies using nsEM at very low ATP concentrations (6, 33), and also to the nsEM data presented here that were obtained at ten times higher ATP concentration than previously.

The “Telemark skier” analogy used to describe the appearance of doubly attached myosin-5a (6) has since been interpreted by some to mean that both motor domains are in an unprimed conformation, with the lead lever bent back to create the knee of the skier (19). The EM data presented here confirms that this is not the case. The leading lever is not bent. Instead, the knee of the leading leg of the skier is actually the lever–motor junction, with the lower leg being formed from the motor domain together with the actin subunit it is attached to and appearing angled backward because the motor is attached to the leading side of the prominent subdomain 1 of actin. In contrast, in the trailing head, actin subdomain 1, the motor domain and the lever are all roughly colinear, creating the different shape resembling the trailing leg of the skier. As the powerstroke of myosin-5a is similar in distance to the canonical step length of 35.75 nm (56), a primed myosin head could easily bind to create the next leading head, with the subsequent rapid release of phosphate (42) generating stress within the doubly attached molecule as the head is unable to adopt the preferred unprimed (postpowerstroke) structure of actomyosin.ADP.

Our nsEM, performed at the same ATP concentration as the iSCAT assay shows that for 11-subunit motor separation, this tethering is insufficient to prevent converter movement to the unprimed position, with concomitant distortion of the leading lever but little change in the trailing lever (Fig. 3*C*). This may explain why we find that the stepping rate is not slower for short steps, but it is unknown whether this also reduces gating of lead motor ADP release.

### Mechanical Properties of Myosin and Impact on ATPase Kinetics.

Our analysis indicates that myosin-5a has to take variable length steps in the iSCAT assay because the sample geometry restricts azimuthal variation, forcing the motor domain to attach to the subunit of the disordered actin filament that is closest to straight ahead. Thus, the mechanical properties of the myosin cannot be directly assessed, only that there is sufficient compliance within the molecule to allow it to vary its step length over the range observed. However, a similar range of step lengths to that found by iSCAT, albeit that 13-subunit steps are more strongly favored, is found by both cryoEM and nsEM, in which the myosin is free to explore around the actin filament to locate binding sites. This similarity indicates that myosin-5a has a preferred lever–lever angle that places the motors ∼36 nm apart, as we previously proposed from iSCAT data (26). The greater frequency of 13-subunit steps in the EM data, unaffected by the change in actin azimuths produced by phalloidin stabilization of F-actin, indicates that when the myosin is free to explore around the actin filament to overcome the effects of CAD, there is a still a preference for binding with a 13-subunit motor separation, reinforcing the idea of a preferred geometry for the singly attached molecule.

The symmetrical Gaussian spread of the frequencies of shorter and longer steps further suggests a thermally driven fluctuation in motor separation that allows both longer and shorter steps. This could arise from bending within the levers and at the lever–motor junctions and from fluctuations in the lever–lever angle. An estimate of the ease of such fluctuations can be derived from the observed spread of step lengths. The SD of the distribution of step lengths leads to an estimate for the stiffness of the molecule along the actin filament axis, through the Equipartition Principle, as stiffness, $\kappa =k_{B}T/\sigma^{2}$. For our nsEM data, σ = 3.289 nm (Table 1) yielding κ=0.374pN/nm. If we suppose that the lever–lever angle is fixed, then since the two heads are mechanically in series, the stiffness of each head would be 0.748 pN/nm. A similar SD (3.0 nm) was reported by Oke (57) also using nsEM, indicating a similar stiffness.

Because we have been able to examine the dependence of stepping kinetics on stride length, we can use this estimate of myosin-5a stiffness to compare our data with earlier optical trap measurements of the impact of external force on kinetics of ADP release and ATP binding by single myosin-5a heads (13, 58, 59). Our EM data indicate that a 13-subunit motor separation has least strain. A 15-subunit separation implies a forward displacement of the lever–lever junction of 2.75 nm, and thus an assisting force on the trailing head of 2.75×0.374=1.03pN. For a 17-subunit separation, the force would be 2.06 pN, which would be expected to produce a marked acceleration of ADP release based on measurements made using optical trapping under different resisting loads (58–60). This should, thus, result in a reduction in B-state dwell time. It remains to be understood why this acceleration was not detected, but we note that in the iSCAT assay the orientation of the head with respect to the actin filament axis is closely specified (and relevant to myosin walking in the cell), whereas in the optical trap, the orientation of the head is not known.

### Implications of Variable Structure of the Stepping Myosin Motor and Its Actin Track.

Our demonstration of variable step and stride lengths for myosin-5a (Fig. 7) provides a framework for understanding how the molecule manages to transport cargoes through the complex and crowded cytoskeletal matrix. The molecule is sufficiently flexible that it can vary its motor separation to accommodate variations in track structure, and even to switch between tracks (26, 61, 62), yet it is stiff enough to move cargo forward. F-actin has intrinsic angular disorder that likely gives it the structural plasticity to fulfill its many cellular roles as single filaments, networks, and bundles through interactions with other proteins, but at the expense of requiring myosins to cope with its disorder. It is tempting to visualize cargo transport in the cell as analogous to railway tracks along which engines are pulling carriages, and thus to imagine actin filaments as fixed tracks with myosin-5 molecules marching robotically along them. Our data show that the nanoscopic reality is less certain and more subtle. Unlike in macroscopic transport systems, both motor and track are “soft,” containing elements of random disorder that create adaptability.

## Materials and Methods

### Protein Purification.

Rabbit skeletal muscle G-actin was prepared using the standard method as described (63) and stored in liquid nitrogen until use. For the iSCAT measurements and nsEM, a mouse myosin-5a HMM-like construct (i.e., having the two heads together with the proximal coiled coil, like heavy meromyosin derived from muscle myosin-2; myosin-5a amino acid sequence 1 to 1,090) with an N-terminal AviTag biotinylation sequence (64, 65) and C- terminal eGFP and FLAG sequence was expressed in the presence of calmodulin, using the Sf9/baculovirus expression system and purified as described previously (66). The amino acid sequence of this construct can be found in *SI Appendix*, Fig. S9. For cryoEM, the melanocyte-specific isoform of full-length mouse myosin-5a heavy chain (67) was expressed together with calmodulin in Sf9 cells, purified using an N-terminal FLAG tag, and stored drop-frozen in liquid nitrogen, similar to as described before (66).

### Description of iSCAT Microscopy.

In iSCAT microscopy (68, 69), a 445 nm diode laser beam is scanned across the sample using a pair of orthogonal acousto-optic deflectors (Gooch & Housego) mapped into the back focal plane of the objective (PlanApo N 60x, 1.42 NA, Olympus) with a 4f telescope, giving an approximately 30 × 30 μm illumination. The illumination and detection paths are separated using a quarter-wave plate beneath the objective and polarizing beam splitter after the 4f telescope. The detected signal is imaged onto a CMOS camera (MV-D1024-160-CL-8, Photonfocus) at approximately 333× magnification (31.8 nm/pixel). The true magnification was determined using a calibrated resolution grid (Thorlabs) (*SI Appendix*, Fig. S1*D*).

### Single Molecule Motility Assay Using iSCAT Microscopy.

Myosin-5a HMM construct was biotinylated and bound to streptavidin-conjugated 20 nm gold nanoparticles. The flow cell was prepared using 50 × 25 and 25 × 25 mm #1.5 coverslips separated with 100 μm double sided tape as described (70, 71). The flow cell was loaded with F-actin and monitored via the iSCAT microscope, as it can monitor label-free proteins (72). After blocking with BSA, a myosin-5 ATP mixture was introduced. The final assay composition in which stepping was observed, was as follows: 77 pM 20 nm gold nanoparticle attached with 25 pM myosin-5a HMM, 10 μM ATP, 5 μM calmodulin, 40 mM KCl, 5 mM MgCl_2_, 0.1 mM EGTA, 5 mM DTT, 20 mM MOPS (pH 7.3). Data collection was performed at room temperature (19 to 22 °C) and flow cells were not used for more than 30 min.

### iSCAT Data Analysis.

iSCAT images were collected at 200 Hz and averaged to 100 Hz. After 2D Gaussian fitting, the x,y positions of the gold particles were used to calculate myosin stride sizes. Binned stride lengths were fit with a sum of seven Gaussian functions using a least-squares method. The probabilities of step lengths were calculated by a least squares best-fit solution to the set of simultaneous equations containing the probabilities of the underlying step lengths. Dwell time models were fit with maximum likelihood functions, minimizing a single global negative log-likelihood function for all strides.

### Electron Microscopy.

For nsEM, F-actin was stabilized with equimolar phalloidin and mixed with the same myosin-5a construct used in the iSCAT assays but without gold beads, in the presence of 10 μM ATP. The final mixture comprised 500 nM F-actin, 25 nM myosin in 50 mM NaCl, 1 mM MgCl_2_, 0.1 mM EGTA, 10 μM ATP, 10 mM MOPS, pH 7.0. After 30 s incubation, samples were negative stained with uranyl acetate on carbon filmed EM grids and imaged by CCD camera at 0.31 nm/pixel. For cryoEM, full-length mouse myosin-5a was incubated with four moles calmodulin per mole myosin overnight in ice. ATP was added, then F-actin, to give a final mixture comprising 0.83 μM myosin-5a, 3.6 μM calmodulin, 2.1 μM F-actin, 0.20 mM ATP, 95 mM KCl, 48 μM MgCl_2_, 0.12 mM EGTA, 10 mM MOPS, pH 7.0 at 22 °C. The mixture was applied within 10 s to a lacey carbon grid (glow discharged in amylamine), quickly blotted with Ca-free paper and flash frozen in ethane slush. Images were recorded at ∼4μm underfocus by CCD camera at 0.589 nm/pixel.

## Supplementary Material

Appendix 01 (PDF)

## Data Availability

Code, data and figures used for this article. Data have been deposited in figshare server. All data can be found on the figshare server at the URL below: Code: https://figshare.com/s/c6ecde3e4a4cd37d95f7 (73). Data: https://figshare.com/s/2af7af2f464d7a42497a (74). Figures: https://figshare.com/s/b339aaf49fc2187abee0 (75). All other data are included in the manuscript and/or *SI Appendix*.

## References

[1] J. A. Hammer 3rd, J. R. Sellers, Walking to work: Roles for class V myosins as cargo transporters. Nat. Rev. Mol. Cell Biol. **13**, 13–26 (2011). 22146746 10.1038/nrm3248

[2] S. Wong, L. S. Weisman, Roles and regulation of myosin V interaction with cargo. Adv. Biol. Regul. **79**, 100787 (2021). 33541831 10.1016/j.jbior.2021.100787 PMC7920922

[3] J. R. Sellers, Y. Takagi, How myosin 5 walks deduced from single-molecule biophysical approaches. Adv. Exp. Med. Biol. **1239**, 153–181 (2020). 32451859 10.1007/978-3-030-38062-5_8

[4] J. R. Sellers, C. Veigel, Walking with myosin V. Curr. Opin. Cell Biol. **18**, 68–73 (2006). 16378722 10.1016/j.ceb.2005.12.014

[5] H. L. Sweeney, A. Houdusse, The motor mechanism of myosin V: Insights for muscle contraction. Philos. Trans. R. Soc. Lond B Biol. Sci. **359**, 1829–1841 (2004). 15647159 10.1098/rstb.2004.1576 PMC1693472

[6] M. L. Walker , Two-headed binding of a processive myosin to F-actin. Nature **405**, 804–807 (2000). 10866203 10.1038/35015592

[7] S. Burgess , The prepower stroke conformation of myosin V. J. Cell Biol. **159**, 983–991 (2002). 12499355 10.1083/jcb.200208172 PMC2173995

[8] J. N. Forkey, M. E. Quinlan, M. A. Shaw, J. E. Corrie, Y. E. Goldman, Three-dimensional structural dynamics of myosin V by single-molecule fluorescence polarization. Nature **422**, 399–404 (2003). 12660775 10.1038/nature01529

[9] M. Rief , Myosin-V stepping kinetics: A molecular model for processivity. Proc. Natl. Acad. Sci. U.S.A. **97**, 9482–9486 (2000). 10944217 10.1073/pnas.97.17.9482 PMC16890

[10] A. D. Mehta , Myosin-V is a processive actin-based motor. Nature **400**, 590–593 (1999). 10448864 10.1038/23072

[11] A. Yildiz , Myosin V walks hand-over-hand: Single fluorophore imaging with 1.5-nm localization. Science **300**, 2061–2065 (2003). 12791999 10.1126/science.1084398

[12] T. Sakamoto, I. Amitani, E. Yokota, T. Ando, Direct observation of processive movement by individual myosin V molecules. Biochem. Biophys. Res. Commun. **272**, 586–590 (2000). 10833456 10.1006/bbrc.2000.2819

[13] C. Veigel, F. Wang, M. L. Bartoo, J. R. Sellers, J. E. Molloy, The gated gait of the processive molecular motor, myosin V. Nat. Cell Biol. **4**, 59–65 (2002). 11740494 10.1038/ncb732

[14] T. Sakamoto, M. R. Webb, E. Forgacs, H. D. White, J. R. Sellers, Direct observation of the mechanochemical coupling in myosin Va during processive movement. Nature **455**, 128–132 (2008). 18668042 10.1038/nature07188 PMC2775414

[15] T. D. Pollard, J. A. Cooper, Actin and actin-binding proteins. A critical evaluation of mechanisms and functions. Annu. Rev. Biochem. **55**, 987–1035 (1986). 3527055 10.1146/annurev.bi.55.070186.005011

[16] T. D. Pollard, Actin and actin-binding proteins. Cold Spring Harb. Perspect. Biol. **8** (2016). 10.1101/cshperspect.a018226 PMC4968159 26988969

[17] M. J. Reynolds, C. Hachicho, A. G. Carl, R. Gong, G. M. Alushin, Bending forces and nucleotide state jointly regulate F-actin structure. Nature **611**, 380–386 (2022). 36289330 10.1038/s41586-022-05366-w PMC9646526

[18] W. Oosterheert, B. U. Klink, A. Belyy, S. Pospich, S. Raunser, Structural basis of actin filament assembly and aging. Nature **611**, 374–379 (2022). 36289337 10.1038/s41586-022-05241-8 PMC9646518

[19] G. E. Snyder, T. Sakamoto, J. A. Hammer 3rd, J. R. Sellers, P. R. Selvin, Nanometer localization of single green fluorescent proteins: Evidence that myosin V walks hand-over-hand via telemark configuration. Biophys. J. **87**, 1776–1783 (2004). 15345556 10.1529/biophysj.103.036897 PMC1304582

[20] T. Sakamoto, A. Yildiz, P. R. Selvin, J. R. Sellers, Step-size is determined by neck length in myosin V. Biochemistry **44**, 16203–16210 (2005). 16331980 10.1021/bi0512086

[21] D. M. Warshaw , Differential labeling of myosin V heads with quantum dots allows direct visualization of hand-over-hand processivity. Biophys. J. **88**, L30–L32 (2005). 15764654 10.1529/biophysj.105.061903 PMC1305523

[22] H. Lu, G. G. Kennedy, D. M. Warshaw, K. M. Trybus, Simultaneous observation of tail and head movements of myosin V during processive motion. J. Biol. Chem. **285**, 42068–42074 (2010). 20974847 10.1074/jbc.M110.180265 PMC3009932

[23] K. Lindfors, T. Kalkbrenner, P. Stoller, V. Sandoghdar, Detection and spectroscopy of gold nanoparticles using supercontinuum white light confocal microscopy. Phys. Rev. Lett. **93**, 037401 (2004). 15323866 10.1103/PhysRevLett.93.037401

[24] V. Jacobsen, P. Stoller, C. Brunner, V. Vogel, V. Sandoghdar, Interferometric optical detection and tracking of very small gold nanoparticles at a water-glass interface. Opt. Express **14**, 405–414 (2006). 19503354 10.1364/opex.14.000405

[25] P. Kukura , High-speed nanoscopic tracking of the position and orientation of a single virus. Nat. Methods **6**, 923–927 (2009). 19881510 10.1038/nmeth.1395

[26] J. Andrecka , Structural dynamics of myosin 5 during processive motion revealed by interferometric scattering microscopy. Elife **4**, e05413 (2015). 25748137 10.7554/eLife.05413 PMC4391024

[27] E. H. Egelman, N. Francis, D. J. DeRosier, F-actin is a helix with a random variable twist. Nature **298**, 131–135 (1982). 7201078 10.1038/298131a0

[28] E. H. Egelman, D. J. DeRosier, Image analysis shows that variations in actin crossover spacings are random, not compensatory. Biophys. J. **63**, 1299–1305 (1992). 1477281 10.1016/S0006-3495(92)81716-2 PMC1261433

[29] A. Orlova, E. H. Egelman, F-actin retains a memory of angular order. Biophys. J. **78**, 2180–2185 (2000). 10733996 10.1016/S0006-3495(00)76765-8 PMC1300810

[30] M. Y. Ali , Myosin V is a left-handed spiral motor on the right-handed actin helix. Nat. Struct. Biol. **9**, 464–467 (2002). 12006986 10.1038/nsb803

[31] J. H. Lewis, J. F. Beausang, H. L. Sweeney, Y. E. Goldman, The azimuthal path of myosin V and its dependence on lever-arm length. J. Gen. Physiol. **139**, 101–120 (2012). 22291144 10.1085/jgp.201110715 PMC3269788

[32] M. E. Arsenault, Y. Sun, H. H. Bau, Y. E. Goldman, Using electrical and optical tweezers to facilitate studies of molecular motors. Phys. Chem. Chem. Phys. **11**, 4834–4839 (2009). 19506758 10.1039/b821861g PMC3639145

[33] O. A. Oke , Influence of lever structure on myosin 5a walking. Proc. Natl. Acad. Sci. U.S.A. **107**, 2509–14 (2010). 20133809 10.1073/pnas.0906907107 PMC2823865

[34] S. Pospich, H. L. Sweeney, A. Houdusse, S. Raunser, High-resolution structures of the actomyosin-V complex in three nucleotide states provide insights into the force generation mechanism. Elife **10**, e73724 (2021). 34812732 10.7554/eLife.73724 PMC8735999

[35] D. N. Krementsov, E. B. Krementsova, K. M. Trybus, Myosin V: Regulation by calcium, calmodulin, and the tail domain. J. Cell Biol. **164**, 877–886 (2004). 15007063 10.1083/jcb.200310065 PMC2172279

[36] F. Wang , Regulated conformation of myosin V. J. Biol. Chem. **279**, 2333–2336 (2004). 14634000 10.1074/jbc.C300488200

[37] J. Liu, D. W. Taylor, E. B. Krementsova, K. M. Trybus, K. A. Taylor, Three-dimensional structure of the myosin V inhibited state by cryoelectron tomography. Nature **442**, 208–211 (2006). 16625208 10.1038/nature04719

[38] K. Thirumurugan, T. Sakamoto, J. A. Hammer 3rd, J. R. Sellers, P. J. Knight, The cargo-binding domain regulates structure and activity of myosin 5. Nature **442**, 212–215 (2006). 16838021 10.1038/nature04865 PMC1852638

[39] R. D. Vale, R. A. Milligan, The way things move: Looking under the hood of molecular motor proteins. Science **288**, 88–95 (2000). 10753125 10.1126/science.288.5463.88

[40] K. C. Holmes, R. R. Schroder, H. L. Sweeney, A. Houdusse, The structure of the rigor complex and its implications for the power stroke. Philos. Trans. R Soc. Lond. B Biol. Sci. **359**, 1819–1828 (2004). 15647158 10.1098/rstb.2004.1566 PMC1693467

[41] P. Knight, J. Trinick, Structure of the myosin projections on native thick filaments from vertebrate skeletal muscle. J. Mol. Biol. **177**, 461–482 (1984). 6540810 10.1016/0022-2836(84)90295-x

[42] S. S. Rosenfeld, H. L. Sweeney, A model of myosin V processivity. J. Biol. Chem. **279**, 40100–40111 (2004). 15254035 10.1074/jbc.M402583200

[43] E. Forgacs , Kinetics of ADP dissociation from the trail and lead heads of actomyosin V following the power stroke. J. Biol. Chem. **283**, 766–773 (2008). 17965414 10.1074/jbc.M704313200

[44] V. E. Galkin, A. Orlova, E. H. Egelman, Actin filaments as tension sensors. Curr. Biol. **22**, R96–R101 (2012). 22321312 10.1016/j.cub.2011.12.010 PMC3277726

[45] V. E. Galkin, A. Orlova, M. R. Vos, G. F. Schroder, E. H. Egelman, Near-atomic resolution for one state of F-actin. Structure **23**, 173–182 (2015). 25533486 10.1016/j.str.2014.11.006 PMC4286464

[46] S. Das , D-loop dynamics and near-atomic-resolution cryo-EM structure of phalloidin-bound F-actin. Structure **28**, 586–593 e3 (2020). 32348747 10.1016/j.str.2020.04.004 PMC7316398

[47] N. Volkmann , The structural basis of myosin V processive movement as revealed by electron cryomicroscopy. Mol. Cell **19**, 595–605 (2005). 16137617 10.1016/j.molcel.2005.07.015

[48] F. Merino , Structural transitions of F-actin upon ATP hydrolysis at near-atomic resolution revealed by cryo-EM. Nat. Struct. Mol. Biol. **25**, 528–537 (2018). 29867215 10.1038/s41594-018-0074-0

[49] S. Z. Chou, T. D. Pollard, Mechanism of actin polymerization revealed by cryo-EM structures of actin filaments with three different bound nucleotides. Proc. Natl. Acad. Sci. U.S.A. **116**, 4265–4274 (2019). 30760599 10.1073/pnas.1807028115 PMC6410863

[50] R. Gong , Structural basis for tunable control of actin dynamics by myosin-15 in mechanosensory stereocilia. Sci. Adv. **8**, eabl4733 (2022). 35857845 10.1126/sciadv.abl4733 PMC9299544

[51] W. Oosterheert , Molecular mechanisms of inorganic-phosphate release from the core and barbed end of actin filaments. Nat. Struct. Mol. Biol. **30**, 1774–1785 (2023). 37749275 10.1038/s41594-023-01101-9 PMC10643162

[52] A. S. Arora , Structural insights into actin isoforms. Elife **12**, e82015 (2023). 36790143 10.7554/eLife.82015 PMC10072879

[53] T. Sakamoto , Neck length and processivity of myosin V. J. Biol. Chem. **278**, 29201–29207 (2003). 12740393 10.1074/jbc.M303662200

[54] J. E. Baker , Myosin V processivity: Multiple kinetic pathways for head-to-head coordination. Proc. Natl. Acad. Sci. U.S.A. **101**, 5542–5546 (2004). 15056760 10.1073/pnas.0307247101 PMC397419

[55] A. R. Hodges, E. B. Krementsova, K. M. Trybus, Engineering the processive run length of Myosin V. J. Biol. Chem. **282**, 27192–27197 (2007). 17640878 10.1074/jbc.M703968200

[56] D. P. Klebl *et al*., Swinging lever mechanism of myosin directly demonstrated by time-resolved cryoem. bioRxiv [Preprint] (2024). http://biorxiv.org/content/early/2024/01/06/2024.01.05.574365.abstract (Accessed 30 January 2024).

[57] O. A. Oke, Electron Microscopy of myosin V molecules. Thesis (2004). https://ethos.bl.uk/OrderDetails.do?uin=uk.bl.ethos.405799.

[58] C. Veigel, S. Schmitz, F. Wang, J. R. Sellers, Load-dependent kinetics of myosin-V can explain its high processivity. Nat. Cell Biol. **7**, 861–869 (2005). 16100513 10.1038/ncb1287

[59] J. R. Sellers, C. Veigel, Direct observation of the myosin-Va power stroke and its reversal. Nat. Struct. Mol. Biol. **17**, 590–595 (2010). 20418880 10.1038/nsmb.1820 PMC3487478

[60] T. J. Purcell, H. L. Sweeney, J. A. Spudich, A force-dependent state controls the coordination of processive myosin V. Proc. Natl. Acad. Sci. U.S.A. **102**, 13873–13878 (2005). 16150709 10.1073/pnas.0506441102 PMC1236568

[61] J. Bao, D. Huck, L. K. Gunther, J. R. Sellers, T. Sakamoto, Actin structure-dependent stepping of myosin 5a and 10 during processive movement. PLoS One **8**, e74936 (2013). 24069366 10.1371/journal.pone.0074936 PMC3777900

[62] B. L. Ricca, R. S. Rock, The stepping pattern of myosin X is adapted for processive motility on bundled actin. Biophys. J. **99**, 1818–1826 (2010). 20858426 10.1016/j.bpj.2010.06.066 PMC2941030

[63] J. A. Spudich, S. Watt, The regulation of rabbit skeletal muscle contraction. I. Biochemical studies of the interaction of the tropomyosin-troponin complex with actin and the proteolytic fragments of myosin. J. Biol. Chem. **246**, 4866–4871 (1971). 4254541

[64] D. Beckett, E. Kovaleva, P. J. Schatz, A minimal peptide substrate in biotin holoenzyme synthetase-catalyzed biotinylation. Protein Sci. **8**, 921–929 (1999). 10211839 10.1110/ps.8.4.921 PMC2144313

[65] T. Lin, M. J. Greenberg, J. R. Moore, E. M. Ostap, A hearing loss-associated myo1c mutation (R156W) decreases the myosin duty ratio and force sensitivity. Biochemistry **50**, 1831–1838 (2011). 21265502 10.1021/bi1016777 PMC3059334

[66] F. Wang , Effect of ADP and ionic strength on the kinetic and motile properties of recombinant mouse myosin V. J. Biol. Chem. **275**, 4329–4335 (2000). 10660602 10.1074/jbc.275.6.4329

[67] X. Wu, T. Sakamoto, F. Zhang, J. R. Sellers, J. A. Hammer 3rd, In vitro reconstitution of a transport complex containing Rab27a, melanophilin and myosin Va. FEBS Lett. **580**, 5863–5868 (2006). 17045265 10.1016/j.febslet.2006.09.047

[68] J. Ortega-Arroyo, P. Kukura, Interferometric scattering microscopy (iSCAT): New frontiers in ultrafast and ultrasensitive optical microscopy. Phys. Chem. Chem. Phys. **14**, 15625–15636 (2012). 22996289 10.1039/c2cp41013c

[69] Y. Takagi, N. Hundt, A. Fineberg, Single-molecule biophysical techniques to study actomyosin force transduction. Adv. Exp. Med. Biol. **1239**, 85–126 (2020). 32451857 10.1007/978-3-030-38062-5_6

[70] A. R. Dunn, J. A. Spudich, Dynamics of the unbound head during myosin V processive translocation. Nat. Struct. Mol. Biol. **14**, 246–248 (2007). 17293871 10.1038/nsmb1206

[71] A. R. Dunn, J. A. Spudich, Single-molecule gold-nanoparticle tracking. Cold Spring Harb. Protoc. **2011**, 1498–1506 (2011). 22135665 10.1101/pdb.prot066977 PMC4799655

[72] J. Ortega Arroyo , Label-free, all-optical detection, imaging, and tracking of a single protein. Nano Lett. **14**, 2065–2070 (2014). 24597479 10.1021/nl500234t PMC4186656

[73] A. Fineberg, Code for “Myosin-5 varies its step length to carry cargo straight along the irregular F-actin track.” Figshare. 10.25444/nhlbi.23681457. Deposited 6 March 2024. PMC10990141 38507449

[74] A. Fineberg, Data for “Myosin-5 varies its step length to carry cargo straight along the irregular F-actin track.” Figshare. 10.25444/nhlbi.23681463. Deposited 6 March 2024. PMC10990141 38507449

[75] A. Fineberg, Figures for “Myosin-5 varies its step length to carry cargo straight along the irregular F-actin track.” Figshare. 10.25444/nhlbi.23681469. Deposited 6 March 2024. PMC10990141 38507449

